# The post COVID-19 healthcare landscape and the use of long-acting injectable antipsychotics for individuals with schizophrenia and bipolar I disorder: the importance of an integrated collaborative-care approach

**DOI:** 10.1186/s12888-022-03685-w

**Published:** 2022-01-10

**Authors:** Christoph U. Correll, Craig Chepke, Paul Gionfriddo, Joe Parks, Phyllis Foxworth, Anirban Basu, Teri S. Brister, Dawn Brown, Christopher Clarke, Youssef Hassoun

**Affiliations:** 1 The Zucker Hillside Hospital, Department of Psychiatry Research, Northwell Health, 75-59 263rd Street, Glen Oaks, NY 11004 USA; 2 Donald and Barbara Zucker School of Medicine at Hofstra/Northwell, Department of Psychiatry and Molecular Medicine, Hempstead, NY USA; 3 Charité Universitätsmedizin Berlin, Department of Child and Adolescent Psychiatry, Berlin, Germany; 4 Excel Psychiatric Associates, Huntersville, NC USA; 5 Mental Health America, Alexandria, VA USA; 6 National Council for Behavioral Health, Washington, DC USA; 7 Depression and Bipolar Support Alliance, Chicago, IL USA; 8 The Comparative Health Outcomes, Policy, and Economics (CHOICE) Institute, Departments of Pharmacy, Health Services, and Economics, University of Washington, Seattle, WA USA; 9 National Alliance on Mental Illness, Arlington, VA USA; 10 Enterprise Digital Health, University of Southern California, Los Angeles, CA USA

**Keywords:** Bipolar I disorder, Collaborative-care, COVID-19, Long-acting injectable antipsychotics, Schizophrenia

## Abstract

**Background:**

Long-acting injectable antipsychotics (LAIs) are an essential maintenance treatment option for individuals with schizophrenia or bipolar I disorder (BP-I). This report summarizes a roundtable discussion on the impact of COVID-19 on the mental healthcare landscape and use of LAIs for individuals with schizophrenia or BP-I.

**Methods:**

Ten experts and stakeholders from diverse fields of healthcare participated in a roundtable discussion on the impact of the COVID-19 pandemic, treatment challenges, and gaps in healthcare for individuals with schizophrenia or BP-I, informed by a literature search.

**Results:**

Individuals with schizophrenia or BP-I are at increased risk of COVID-19 infection and increased risk of mortality after COVID-19 diagnosis. LAI prescriptions decreased early on in the pandemic, driven by a decrease in face-to-face consultations. Mental healthcare services are adapting with increased use of telehealth and home-based treatment. Clinical workflows to provide consistent, in-person LAI services include screening for COVID-19 exposure and infection, minimizing contact, and ensuring mask-wearing by individuals and staff. The importance of continued in-person visits for LAIs needs to be discussed so that staff can share that information with patients, their caregivers, and families. A fully integrated, collaborative-care model is the most important aspect of care for individuals with schizophrenia or BP-I during and after the COVID-19 pandemic.

**Conclusions:**

The COVID-19 pandemic has highlighted the importance of a fully integrated collaborative-care model to ensure regular, routine healthcare contact and access to prescribed treatments and services for individuals with schizophrenia and BP-I.

## Background

It is currently estimated that 0.3–0.6% of adults in the United States have schizophrenia (SCZ), and each year about 2.8% have bipolar I disorder (BP-I) [1, 2]. Individuals with SCZ or BP-I have a disease onset in the late teens and early twenties [3] and an increased risk of premature death compared with the general population, including increased cardiovascular mortality and a higher risk of death by suicide [4–7]. Among other major disruptions worldwide, the COVID-19 pandemic has significantly changed the access to and provision of physical and mental healthcare [8, 9].

People with SCZ, BP-I, or other severe mental illnesses have been disproportionally affected by the COVID-19 pandemic [10–13]. Illness characteristics (e.g., psychosis and cognitive dysfunction) and sociodemographic characteristics (e.g., living in group housing, being homeless) may impact pandemic-related social distancing measures in individuals with SCZ or BP-I, thus increasing infection risk [10]. In addition, illness- and/or treatment-related physical comorbidities and low socioeconomic status may increase the risk of adverse health outcomes of the COVID-19 infection in individuals with SCZ or BP-I compared with the general population [10].

Many individuals with SCZ or BP-I require long-term treatment with antipsychotics to prevent illness relapse and maintain health-related quality of life, and long-acting injectable antipsychotics (LAIs) are an essential maintenance treatment option for these people [14]. Compared with oral antipsychotics, LAIs are associated with significantly reduced all-cause discontinuation, relapse, and hospitalization [15], and significantly lower overall mortality [16]. Current American Psychiatric Association (APA) treatment guidelines for individuals with SCZ support the use of LAIs if a person prefers this treatment option or if they have a history of poor or uncertain adherence [17]. Several other SCZ guidelines recommend offering LAIs to individuals experiencing their first-episode, and before non-adherence or relapse have occurred [18, 19]. Despite these recommendations and their clinical effectiveness, LAIs are underutilized, with only 13 to 28% of all eligible individuals in the United States receiving an LAI [14].

It was reported that the administration of LAIs was suspended in some areas during the COVID-19 pandemic because it was considered an elective procedure [20, 21], prompting the APA to issue specific COVID-19 pandemic guidance on the administration of LAIs [21]. In this guidance, the APA encourages clinics, hospitals, and other medical facilities to include the ongoing use of LAIs for people with high-risk chronic illness as a necessary procedure during the COVID-19 pandemic, noting that treatment withdrawal would likely increase the risk of physical and psychiatric decompensation [21]. Clinical considerations for LAI administration during the COVID-19 pandemic include whether an individual with a psychotic illness should receive LAI therapy, which LAI should be administered, when and where it should be administered, and what safety measures should be implemented to minimize COVID-19 exposure risk [22–24].

The aim of this report was to summarize results from a roundtable discussion that took place on March 26, 2021, during which 15 experts and stakeholders from diverse fields of healthcare reviewed data from a systematic literature search and discussed the impact of COVID-19 on the mental healthcare landscape, the long-term impact of the COVID-19 pandemic on clinical practice, and the long-term management of individuals with SCZ or BP-I within this changing landscape.

## Methods

A comprehensive literature search that provided the basis for an informed discussion as part of a roundtable was performed, with the search strategy developed using a combination of Medical Subject Heading (MeSH) terms and keywords. The search was conducted across the PubMed, OVID Medline, and CINAHL databases, with PubMed as the primary literature database to structure the search criteria (Table 1). The search was conducted on March 10, 2021, to identify any available scientific literature related to the research question. Two independent researchers conducted the search and extracted information from the articles, first by reviewing titles and abstracts, and then by reviewing the full text articles. All identified evidence was reviewed by the first author and shared with the authorship team to facilitate discussion.

**Table 1:** Literature search criteria

Parameter	Details
Research Question	“How has the COVID-19 pandemic impacted the ability of adult individuals diagnosed with schizophrenia or bipolar I disorder to access appropriate treatment, including the use of a long-acting injectable antipsychotic?”
Individual Population	Adults aged ≥18 years. Literature Database Categories: Adult (18+ years); Young Adult (19–24 years); Adult (19–44 years); Middle Aged (45–64 years); Aged (65+ years); 80 and over (80+ years)
Therapeutic Indication	Schizophrenia; Bipolar I Disorder
Journal Type	Peer-reviewed
Language	English
Types of Evidence	Case Report; Observation Study; Clinical Study; Clinical Trial; Randomized Controlled Trial; Multicenter Study; Observational Study; Meta-analysis; Systematic Review
Literature Databases	PubMed; OVID Medline; CINAHL
Literature Type	Full text; Free Full Text; Open Access
Time Period	January 1, 2020 – March 10, 2021

A virtual roundtable meeting was held on March 26, 2021, which included a multidisciplinary panel of 10 people, including healthcare professionals, patient representatives/caregivers, payers, policy and advocacy representatives, and a telehealth expert. The meeting opened with a discussion of the identified literature on the incidence of COVID-19 infection rates and relevant outcomes in individuals with SCZ or BP-I, access to treatment during the COVID-19 pandemic, and APA guidance on LAI utilization, focusing on the following key questions:

1. How has the COVID-19 pandemic impacted individuals with SCZ or BP-I, and how may this change post-pandemic?
2. What is the impact of the COVID-19 pandemic on treatment challenges for individuals with SCZ or BP-I, and how these will evolve post COVID-19 pandemic, specifically in the context of LAIs?
3. What gaps exist in the access to healthcare during and after the COVID-19 pandemic and the utility of LAIs?

Further discussion took place to consider what the current needs and opportunities are in mental healthcare resulting from the COVID-19 pandemic.

This report reflects the views of the authors and the published literature and aims to raise awareness of the long-term impact of the COVID-19 pandemic for individuals with SCZ or BP-I, including treatment challenges, healthcare access gaps, current needs, and practical guidance regarding the long-term management of people with serious mental illness.

## Results

We first present results from the systematic literature review that addresses each of the three primary questions that were posed to the roundtable participants, then summarize the identified issues and provide an overview of the recommended solutions.

## Literature review

The literature search sought to answer the primary question of *“*How has the COVID-19 pandemic impacted the ability of adult individuals diagnosed with SCZ or BP-I to access appropriate treatment, including the use of a long-acting injectable antipsychotic?” The key points that were identified from the literature review are summarized in Table 2.

**Table 2:** Summary of key points from the literature review

Question	Key literature review results
How has the COVID-19 pandemic impacted the individual with SCZ or BP-I?	Individuals recently diagnosed with SCZ or BP-I are at increased risk of COVID-19 infection, compared with individuals without a mental disorder [25]. A SCZ spectrum diagnosis is associated with mortality after COVID-19 diagnosis [26]. Mental health problems are likely to remain increased beyond the actual pandemic [27].
What is the impact of the COVID-19 pandemic on treatment challenges for individuals with SCZ and BP-I?	The start of the COVID-19 pandemic resulted in reduced access to services, early psychiatric discharge, and disruption to face-to-face psychiatric care for people with pre-existing mental illness, potentially increasing relapse and suicide risk [9].
What are the main gaps to the access of healthcare during and after the COVID-19 pandemic and the utility of LAIs?	LAI prescriptions decreased at the start of the COVID-19 pandemic, driven by a decrease in face-to-face consultations as part of pandemic-related physical distancing measures. Individuals require the most consistent (and convenient) access to LAIs [20, 21].

*BP-I* bipolar I disorder, *LAI* long-acting injectable antipsychotics, *SCZ* schizophrenia

### How has the COVID-19 pandemic impacted individuals with SCZ or BP-I, and how may this change post-pandemic?

A large U.S. electronic health records study showed that individuals recently diagnosed with a mental disorder were at significantly increased risk of COVID-19 infection. Adjusted odds ratios were 9.89 for people with SCZ and 7.69 for those with BP-I, compared to those without a mental disorder [25]. Findings from another large health records study showed that, among people diagnosed with COVID-19 infection, overall 33.6% received a neurological or psychiatric diagnosis in the following 6 months, including 12.8% who received their first recorded neurological or psychiatric diagnosis—substantially more than in the comparator cohort with influenza [28]. Mental health problems and suicidal behavior that are exacerbated by the pandemic are likely to remain increased beyond the actual pandemic [27]. Psychiatric emergency admissions decreased at the start of the COVID-19 pandemic lockdown, but the percentage of individuals hospitalized for acute psychiatric care increased significantly [29]. An SCZ spectrum diagnosis was significantly associated with mortality in the 45 days after a positive COVID-19 diagnosis in a U.S. cohort study [11, 25, 26]. Data from a French national hospital database showed higher in-hospital mortality and lower intensive care unit admission rates for individuals with SCZ than for matched controls; differences were statistically significant in the ≥65-years to <80-years age group [30].

### What is the impact of the COVID-19 pandemic on treatment challenges for individuals with SCZ or BP-I, and how these will evolve post-pandemic, specifically in the context of LAIs?

The fact that people with serious mental illness are at increased risk of COVID-19 infection—and have higher rates of mortality—means that it is important to prioritize COVID-19 vaccination in this group of individuals [31]. Vaccination in individuals with SCZ or BP-I may be hampered by negative beliefs and misconceptions about the vaccine’s safety; uptake can be increased with education by healthcare professionals [31]. The start of the COVID-19 pandemic resulted in reduced access to services, early psychiatric discharge, and disruption to face-to-face psychiatric care for people with pre-existing mental illness, potentially increasing relapse and suicide risk [9]. The number of general telehealth visits increased by 50% during the first quarter of 2020 compared with the same period in 2019 [32]. Mental health conditions were the most common telehealth diagnoses, making up about 50% of U.S. telehealth claims in January 2021 [33], up from 30% in January 2020 [34]. Psychotherapy accounted for two of the top five procedure codes by utilization post-pandemic [33].

### What are the main gaps to the access of healthcare during and after the COVID-19 pandemic and the utility of LAIs, and what are the solutions to close those gaps?

The use of LAIs decreased at the start of the COVID-19 pandemic, driven by a decrease in face-to-face consultations as part of pandemic-related physical distancing measures [20, 21]. Additionally, many institutions and healthcare providers viewed LAIs as an elective procedure, which was restricted in many states and healthcare systems [35–38]. Some healthcare providers and medical centers were, however, able to maintain the administration of LAIs after COVID-19 pandemic–related restrictions were introduced, for example, by implementing new approaches, such as office-based drive-up / walk-through clinics to administer injections, as well as at-home service providers [22, 39]. Increased use of telepsychiatry nurse visits and clozapine mailing or curbside pick-up ensured continuity of care for individuals with SCZ treated at an ambulatory clinic [40].

During the roundtable discussion, three main gaps in the mental healthcare of individuals with SCZ or BP-I, as well as potential solutions to address these gaps, were identified. These gaps related mainly to (1) the increased risk for COVID-19 infection and sequelae, (2) decreased access to psychiatric care, and (3) inconsistent management of LAI maintenance care (Table 3). Solutions identified by the roundtable participants focused on elements such as integrated collaborative care, increased use of telehealth and home-based treatment options, and implementation of clinical workflows to provide consistent, in-person LAI injection services (Table 3). Mental health services implemented infection control measures, including screening patients for COVID-19 infection, minimizing contact, and ensuring mask-wearing by all individuals and staff [9, 22]. Highlighting the importance of in-person visits for LAIs, so that staff can share that information with individuals, their caregivers, and families, helps support continued access to LAI maintenance treatment [22]. There are potential positive effects of COVID-19 pandemic-related healthcare service changes, such as reassessment and expanded reimbursement of telehealth and at-home treatment options, increased acceptance of LAIs, and an increased focus on shared decision making [9].

**Table 3:** Summary of issues identified and recommended solutions

Issue	Solution
Individuals with SCZ, BP-I, or other severe mental illness are at increased risk of COVID-19 infection and associated morbidity and mortality.	A fully integrated, collaborative-care model is the most important aspect of care for individuals with SCZ or BP-I during and after the COVID-19 pandemic. It is important to prioritize COVID-19 vaccination in individuals with serious mental illness [31].
COVID-19 infection control measures have led to reduced access to out-patient, in-hospital, and group-based psychiatric care for individuals with severe mental illness.	Mental healthcare services are adapting to facilitate access to care with increased use of telehealth and home-based treatment [9]. Potential positive effects of COVID-19 pandemic-related healthcare service changes include expanded reimbursement of telehealth and at-home treatment options, increased acceptance of LAIs, and an increased focus on shared decision making [9].
Maintenance of LAI treatment for individuals with severe mental illness has been managed inconsistently within healthcare.	APA guidance encourages ongoing use of LAIs for people with high-risk chronic illness as a necessary procedure during the COVID-19 pandemic [21]. Implementing clinical workflows to provide consistent, in-person LAI injection services includes: screening individuals for COVID-19 exposure and infection, staggering individual appointment times, supplying hand sanitizer, rearranging and expanding the individual waiting area to ensure social distancing and expand individual capacity, ensuring that injecting nurses wear masks and gloves, providing masks for individuals, and discussing the importance of continued in-person visits for LAIs so that staff can share that information with individuals, their caregivers, and families [22].

*APA* American Psychiatric Association, *BP-I* bipolar I disorder, *LAI* long-acting injectable antipsychotics, *SCZ* schizophrenia

## Discussion

The COVID-19 pandemic has taken an outsized toll on the physical and mental health of individuals with SCZ, BP-I, and other severe mental illnesses [9, 13, 22]. A multidisciplinary panel hosted a roundtable discussion to review the literature and discuss personal experiences on the impact of the COVID-19 pandemic on the mental healthcare landscape, clinical practice, and long-term management of individuals with SCZ or BP-I. Based on our roundtable discussion, we identified fully integrated, collaborative care in the delivery of healthcare services as the most important aspect of care for individuals with SCZ or BP-I during and after the COVID-19 pandemic.

The team-based, multidisciplinary approach of the collaborative-care model provides coordinated mental healthcare to implement the appropriate treatment plan [41]. However, to date most collaborative care initiated in primary care has focused on depression and anxiety, not bipolar disorders or schizophrenia. Integrating all aspects of patient care should be extended to people with severe mental illness to ensure they also continue to have access to their individual level of physical and mental healthcare during and post pandemic, including access to prescription treatment, such as LAIs. Greater support of primary care and its closer integration with secondary care could help maintain the provision of mental healthcare during and after the pandemic [9]. Discussions around starting an LAI should be initiated early in the treatment journey and a potential long-term LAI transition considered when initiating oral medications [19]. Important decision points when prescribers may consider introducing LAIs as a treatment option include when an individual is newly diagnosed, has recently relapsed, or is transitioning from in-patient care or incarceration [19]. Some people with mental health disorders have no or poor access to care [42]. Therefore, psychiatrists may need to step up to provide some general medical services, including screening for general medical conditions, counseling on cardiovascular risk reduction, and treating adverse health behaviors such as smoking [42].

The focus on physical healthcare necessitated by COVID-19 infection-control measures has resulted in a contraction of mental healthcare services, with reduced access to out-patient appointments, group psychotherapy, and in-hospital psychiatric care [9]. In response, mental healthcare services are adapting to facilitate access to care with increased use of telehealth and home-based treatment [9]. The uptake of telehealth, including telepsychiatry, has increased markedly since the start of the pandemic [32, 33]. Practices have had to adapt quickly. We have found that having the telehealth consultation separate from the LAI administration visit, with virtual follow-up after the first few injections, is a feasible approach. We anticipate that telepsychiatry will be an important part of the future landscape. A significant investment in human resources to train, manage, and support the ongoing virtual care initiative will be required due to the complex technological configuration needed to deliver a virtual healthcare encounter [43]. Post-pandemic, practices are likely to maintain a hybrid model that will address the best needs for individuals based on a collaborative-care approach that includes the individual and caregiver in decision making. We recommend employing a trauma-informed approach to individual patient care. The majority of people who experience mental health issues, substance abuse conditions, or homelessness have a history of trauma [19]. Trauma-related symptoms may be triggered by the discussion or administration of LAIs in individuals who have a history of trauma, including those who have in the past had medication injections involuntarily administered [19]. A trauma-informed approach includes establishing a trusting relationship and highlighting choice and preference for LAIs [19]. Shared decision making and motivational interviewing are approaches that can promote effective communication and collaboration, supporting patient choice and empowerment [19].

We emphasize the need for the administration of LAIs to be seen as part of the integrated care model, potentially—or even preferably—administered as part of a separate visit independent from psychiatric consultation to destigmatize the injection. We advise that individuals who are deemed eligible for treatment with an LAI should continue to receive their LAI medication. Continuity of care is important to reduce the risk of decompensation in individuals with severe mental illness [21]. Sudden changes to healthcare delivery could increase the risk of patient disengagement, treatment non-adherence, and distress [10]. For individuals with SCZ or BP-I, the disruption of routines and relationships can cause significant stress and a sense of loss, which may lead to a reemergence of symptoms. People with SCZ or BP-I may also experience significant levels of anxiety and depression related to the pandemic and should be assessed for these. Practice providers, especially those who work closely with peer specialists, are well placed to ensure that the individual’s care model remains as normalized as possible during and after the pandemic to maintain LAI treatment and other regular, routine aspects of care. In addition to the traditional in-office setting, various models of collaborative care enable people with SCZ or BP-1 to continue to receive their LAI medication at pharmacies, mental health clinics, drive-through clinics, in-home services, or injection clinics. Organizational support includes educating all members of the healthcare team about the potential benefits of LAIs and how best to talk about these benefits with individuals and their families: medication adherence and the potential benefits of LAIs should be discussed regularly [19]. Barriers to treatment can be addressed by organizational, policy, and procedural updates, for example, by having the individual’s pharmacy payment assistance needs handled by a nurse or pharmacist, arranging transportation to injection appointments, and involving peer specialists with LAI experience as educators on the collaborative-care team. Switching from LAI therapy to reduce potential in-office exposure during the pandemic should be viewed as a choice of last resort. To ensure continuity of care while prioritizing patient and staff safety during the pandemic, recommendations include the development of new protocols which include guidance for patients on safely approaching LAI clinics and screening for COVID-19 symptoms by phone prior to arrival and guidance for patients and staff on use of proper personal protective equipment against COVID-19 [44].

We propose that the full healthcare team, including the individual and caregiver, should be involved in discussions around LAIs. Framing the discussions toward common patient-focused goals, such as taking fewer pills or a lower total amount of antipsychotic medication over time, are benefits that each individual can own, be responsible for, and acknowledge as their own goals and values will bring focus to what is motivating for individuals. Further, we recognize the increasing importance of family members and caregivers as integral parts of the support network of people with severe mental illness during and after the COVID-19 pandemic and the potential of their inclusion into the collaborative-care model. Other aspects to form part of the collaborative-care model may include individual education, disease awareness, and destigmatization of severe mental illness.

## Conclusion

The COVID-19 pandemic has highlighted the importance of a fully integrated collaborative-care model to ensure regular, routine healthcare contact and access to prescribed treatments and services for individuals with SCZ and BP-I, as well as other severe mental disorders.

## Data Availability

Not applicable.

## Abbreviations

APA: American Psychiatric Association
BP-I: Bipolar I disorder
LAI: Long-acting injectable antipsychotics
SCZ: Schizophrenia
